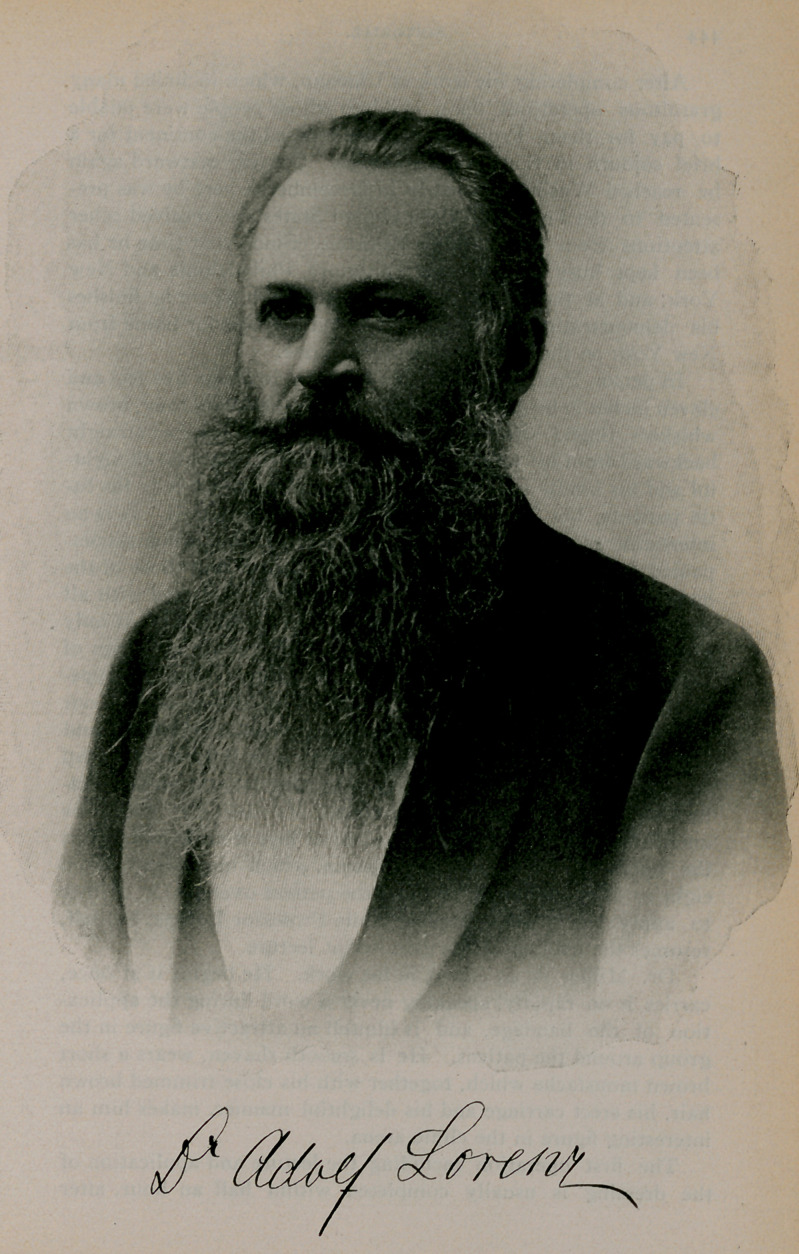# Dr. Adolf Lorenz

**Published:** 1903-01

**Authors:** 


### Dr. Adolf Lorenz.

THE most important medical event of the late autumn and early winter season of 1902, has been and at the present writing continues to be the visit of Professor Dr. Lorenz, the distinguished Vienna orthopedic surgeon, to the principal cities of the United States, in which he has held and is holding clinics for the demonstration of his special work.

When Professor Lorenz arrived at Chicago, in October, for the purpose of operating upon the little daughter of Mr. J. Ogden Armour, the newspapers were filled with sensational accounts of the man and his work. This served in a measure to develop prejudice in the minds of a large portion of the medical profession. It was not long, however, before it was discovered that the distinguished visitor was not responsible for the gratuitous advertising which the "yellows" gave him. His whole mien and bearing was observed to be that of the modest gentleman, which he is. He has not even turned aside to notice these sensational accounts of his tour and work which, in and of itself, is the best evidence of his modesty and sterling worth.

When the Illinois State Board of Health asked Dr. Lorenz to comply with the law and take out a state license, he at once submitted to the necessary examination and courteously thanked the board for calling his attention to the matter. This is in marked contrast to the attitude of some of our American physicians toward the state examination.

Dr Adolf Lorenz

*From the New York Medical Journal.*

After completing his work at Chicago, which included many gratuitous operations upon children whose people were unable to pay for them, Professor Lorenz crossed the continent for a brief sojourn in Southern California. Coming eastward again he reached Washington early in December, where he was presented to the President of the United States and received other attentions from the Austrian Minister. Since that time he has been kept busy operating in Baltimore, Philadelphia and New York, and at the present writing is in Boston where he finishes his demonstrations in this country and will sail for home from New York on the last day of the year 1902.

In person Prof. Lorenz stands erect, he is about five feet and eleven inches in height, is 49 years of age, he wears long, brown whiskers tinged with gray, and brown hair brushed straight backward from his forehead. His manner is calm and thoughtful and his kindly eye and gentle demeanor win not only his little patients, but his attendants and audience as well. It is an interesting picture to see him walk into the clinic room accompanied by his assistant, Dr. Friedreich Müller, and those of the hospital staff who are assigned to work with him, and with all the modesty of a woman begin the toilsome operation that only a stalwart man is equal to deal with. But after the greetings of applause have subsided he at once begins the reading of a typewritten lecture, evidently timid of his ability to master our language extemporaneously, though he speaks it accurately and with only a minimum of foreign accent. His lecture is devoted to a description of his method of operating for congenital luxation of the hip-joint. He continues its delivery until the anesthetised patient is rolled in, when he lays it aside and begins the operation. After its completion, which may occupy from eight to fifteen minutes, he turns the patient over to Dr. Müller to apply the plaster dressing while Professor Lorenz, himself, resumes the reading of his manuscript lecture.

Dr. Müller is an expert in his work. He begins it at once, carries it on rapidly, speaking never a word during the application of the bandage, and is himself an attractive figure in the group around the patient. He is smooth shaven, wears a short brown moustache which, together with his close-trimmed brown hair, his erect carriage and his delightful manner, makes him an interesting figure in the clinic arena.

The first operation including the lecture and application of the dressing is usually completed within half an hour, after which the next patient is rolled in and the process is repeated this time, however, without the reading from the manuscript, though the operator frequently makes remarks explaining the application of the method to the particular case in hand; and this goes on for the third or fourth time, or until both the allotted time and the operators are exhausted.

During his visit to New York, Professor Lorenz held clinics at the Hospital for Ruptured and Crippled, at the Polyclinic, at the Post-Graduate, at Cornell University, at Bellevue, at the New York Orthopedic Dispensary and Hospital, at the King's County Hospital and at the State Hospital for Ruptured and Crippled Children at Tarrytown. These together with his many social engagements served to make a busy week for both himself and Dr. Müller.

We have not attempted a description of the operation itself, in its scientific aspects, because that has been done already by many writers; our aim rather having been to present a pen picture of the man himself at his work. The portrait we publish is an excellent likeness, and will be appreciated by our readers for its artistic merit, we feel sure. It was kindly loaned to us by our esteemed contemporary, *The New York Medical Journal.*

Professor Lorenz has followed wherever the profession has led him, never for a moment pushing himself forward or shrinking from responsibility, but accepting each duty as it presented itself and discharging it with all the zeal and ability of a man who thoroughly understands his work and has an assured faith in its value to mankind. His visit to this country, and especially to New York, will ever be a pleasant memory to those who met him or witnessed his demonstrations of operative skill.

## Figures and Tables

**Figure f1:**